# Persistent Dizziness and Time-Domain Dissociation in Vestibular Function: A Hypothesis-Generating Case Series and Spatiotemporal Framework for Targeted Vestibular Rehabilitation

**DOI:** 10.3390/healthcare14111560

**Published:** 2026-06-03

**Authors:** Leonardo Manzari, Maria Sofia Manzari

**Affiliations:** Msa Ent Academy Center, 03043 Cassino, Italy; mariasofiamanzari@gmail.com

**Keywords:** persistent dizziness, residual dizziness, vestibular compensation, velocity storage, vestibular rehabilitation, time-domain dissociation, caloric testing, rotational testing, otolithic function, visual–vestibular integration, PPPD

## Abstract

**Highlights:**

**What are the main findings?**

- Persistent dizziness may arise from a dissociation between preserved transient/high-frequency vestibular responses and impaired sustained/low-frequency or integrative vestibular processing.
- Four illustrative cases showed distinct patterns of vestibular-domain dissociation, including low-frequency integrative dysfunction, incomplete compensation of unilateral vestibular asymmetry, selective otolithic loss, and bilateral sustained vestibular hypofunction.

**What are the implications of the main findings?**

- Normal or near-normal vHIT findings should not be interpreted as excluding clinically meaningful vestibular dysfunction in patients with persistent dizziness.
- A multidomain vestibular assessment may help identify the specific physiological substrate of persistent symptoms and guide individualized, domain-specific vestibular rehabilitation.

**Abstract:**

**Background/Objectives**: Persistent dizziness after the apparent resolution of an acute or episodic vestibular disorder remains a frequent and clinically challenging condition. In many patients, symptoms persist despite negative positional testing, absence of spontaneous nystagmus, and preserved high-frequency vestibular responses on video head impulse testing. This discrepancy suggests that persistent dizziness may not always be explained by incomplete recovery of a single peripheral vestibular lesion but may reflect a dissociation between transient/high-frequency vestibular responses and sustained/low-frequency or integrative vestibular processing. The aim of this study was to propose a hypothesis-generating, case-based clinical framework for interpreting this dissociation and its implications for targeted vestibular rehabilitation. **Methods**: This was a retrospective, hypothesis-generating, case-based clinical study derived from routine specialist neuro-otological practice. Four illustrative cases were selected because they represented distinct patterns of persistent dizziness in which preserved or near-preserved transient vestibular responses coexisted with abnormalities in sustained, otolithic, visual–vestibular, or velocity-storage-dependent processing. All patients underwent detailed clinical history assessment, bedside neuro-otological examination, and multidomain vestibular assessment according to clinical indication. The purpose of the study was not to estimate prevalence, validate diagnostic accuracy, or demonstrate treatment efficacy but to illustrate a physiology-based interpretive framework. **Results**: The four cases showed different patterns of time-domain dissociation. These included low-frequency integrative dysfunction without clear peripheral lateralization, incompletely compensated unilateral vestibular asymmetry, selective unilateral otolithic loss despite preserved semicircular canal high-frequency responses, and bilateral sustained vestibular hypofunction unmasked by an apparently resolved BPPV-like event. Across cases, persistent symptoms were better explained by the relationship between transient and sustained vestibular domains than by any single test result considered in isolation. **Conclusions**: Persistent dizziness may arise from different combinations of preserved transient vestibular responses and impaired sustained or integrative vestibular processing. The proposed framework does not introduce new vestibular tests and does not claim to validate a new diagnostic entity. Rather, it organizes established vestibular investigations within a time-domain model that may help identify clinically meaningful dissociations and guide individualized, domain-specific vestibular rehabilitation. Prospective studies with larger samples and external validation are required to determine the diagnostic and therapeutic value of this approach.

## 1. Introduction

Persistent dizziness after the apparent resolution of an acute or episodic vestibular disorder remains a frequent and clinically challenging condition. This problem is commonly encountered after benign paroxysmal positional vertigo (BPPV), acute unilateral vestibulopathy, or other vestibular events, when patients continue to report instability, motion sensitivity, visual dependence, or non-specific dizziness despite the disappearance of the original acute signs. In many cases, positional testing becomes negative, spontaneous nystagmus is absent, and high-frequency vestibular testing may appear normal or near-normal. This creates a diagnostic gap between the patient’s persistent symptoms and the apparent normalization of conventional vestibular findings [1,2].

Residual dizziness after BPPV has often been interpreted as a transient, non-specific, or benign post-event phenomenon. However, persistent symptoms may reflect different physiological substrates. Some patients may have incomplete compensation after a previous unilateral vestibular lesion; others may show selective otolithic dysfunction, abnormal visual–vestibular interaction, or reduced low-frequency vestibular responsiveness. In still other cases, an apparently resolved vestibular event may unmask a previously compensated bilateral or integrative vestibular disorder. Therefore, persistent dizziness should not be considered a unitary condition, nor should it be interpreted only on the basis of a single vestibular test [1,2].

A major limitation of routine clinical reasoning is the tendency to equate normal high-frequency vestibular responses with global vestibular recovery. The video head impulse test (vHIT) provides essential information on high-acceleration, high-frequency semicircular canal function, and vestibular-evoked myogenic potentials (VEMPs) provide clinically useful information on transient otolithic pathways. Nevertheless, these tests do not fully explore sustained, low-frequency, visual–vestibular, and velocity-storage-dependent mechanisms. Conversely, caloric testing, rotational chair testing, optokinetic responses, post-rotatory behavior, head-shaking responses, and other dynamic paradigms may disclose abnormalities that are not captured by high-frequency testing alone [3,4].

The distinction between transient and sustained vestibular processing provides a useful physiological basis for this clinical dissociation. High-frequency, jerk-rich stimuli preferentially probe rapid vestibular responses, whereas low-frequency or sustained stimuli more strongly engage central integrative mechanisms, including the velocity storage network. Velocity storage is not merely a prolongation of canal responses; it contributes to the temporal integration, spatial reorientation, and gravity-referenced organization of vestibular signals. Dysfunction within this system may therefore contribute to persistent dizziness, motion sensitivity, and impaired postural–perceptual stability even when direct high-frequency reflex pathways appear preserved [3,5].

The purpose of the present study was not to introduce new vestibular tests or to statistically validate a new diagnostic entity. Rather, we propose a hypothesis-generating, case-based clinical framework in which established vestibular investigations are interpreted according to the temporal domain they preferentially interrogate. In this framework, the clinically relevant finding is not the isolated abnormality of a single test but the pattern of agreement or dissociation between transient/high-frequency responses and sustained/low-frequency or integrative vestibular processing [6,7,8,9,10].

**Figure 1:**
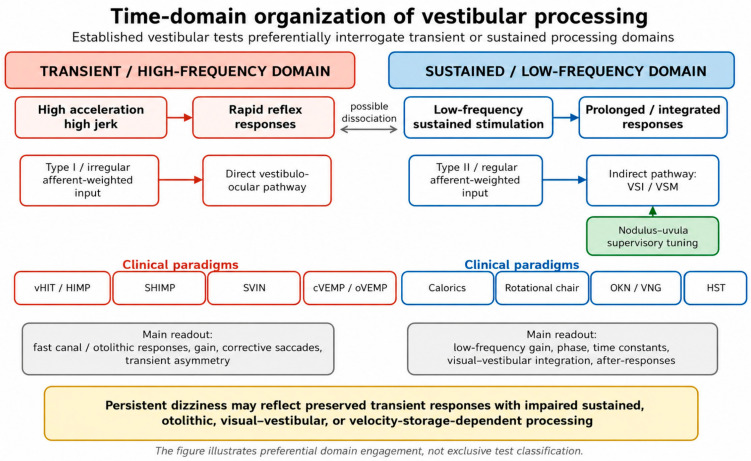
Time-domain organization of vestibular processing. High-frequency, high-acceleration stimuli preferentially engage transient vestibular responses and direct vestibulo-ocular pathways, whereas low-frequency, sustained, rotational, caloric, and visual–vestibular paradigms more strongly engage sustained vestibular processing, multisensory integration, and velocity-storage-dependent mechanisms. The figure does not propose new vestibular tests but illustrates how established clinical paradigms may preferentially interrogate different temporal domains of vestibular function [3,5,6,8,9,10].

To illustrate this concept, we present four representative clinical cases of persistent dizziness selected from routine specialist neuro-otological practice. These cases were chosen because they exemplify distinct patterns of vestibular-domain dissociation: low-frequency integrative dysfunction without clear peripheral lateralization, incompletely compensated unilateral vestibular asymmetry, selective unilateral otolithic loss despite preserved semicircular canal high-frequency responses, and bilateral sustained vestibular hypofunction unmasked by an apparently resolved BPPV-like event. The broader clinical implication is that vestibular rehabilitation should not be prescribed generically but should be tailored to the physiological domain that is primarily dysfunctional [7] (Figure 1).

## 2. Materials and Methods

### 2.1. Study Design

This study was designed as a retrospective, hypothesis-generating, case-based clinical study derived from routine specialist neuro-otological practice. The aim was not to perform a statistically powered cohort analysis, estimate prevalence, test diagnostic accuracy, or demonstrate treatment efficacy. Rather, the objective was to illustrate a physiology-based interpretive framework for persistent dizziness, based on the comparison between transient/high-frequency vestibular responses and sustained/low-frequency or integrative vestibular processing.

The manuscript integrates established concepts from vestibular physiology, clinical neuro-otology, and vestibular rehabilitation with four illustrative clinical observations. These cases were used to exemplify clinically meaningful patterns of dissociation across vestibular domains and to generate hypotheses for future prospective studies.

### 2.2. Clinical Setting and Data Source

The clinical observations reported in this manuscript were derived from routine diagnostic assessments performed at MSA ENT Academy Center, Cassino, Italy, a specialist clinical neuro-otology center dedicated to the assessment, diagnosis, and rehabilitation of vestibular disorders.

All patients were evaluated in a licensed clinical setting as part of standard specialist neuro-otological care. The clinical assessment and patient management were performed by Leonardo Manzari, MD. The data were not obtained from an open database or from a third-party source. They were derived from the clinical records of patients evaluated in routine practice and were retrospectively reviewed in anonymized form for the purpose of developing the present hypothesis-generating clinical framework. No experimental intervention was performed for research purposes. The vestibular tests reported in the manuscript were performed because they were considered clinically appropriate for the diagnostic assessment of each patient.

### 2.3. Author Roles in Clinical Assessment and Data Handling

Clinical assessment, diagnostic interpretation, vestibular data interpretation, diagnosis, and patient management were performed by Leonardo Manzari, MD.

Instrumental vestibular and neurophysiological tests were performed in routine clinical practice by qualified clinical personnel according to their professional competencies. Maria Sofia Manzari holds a degree in Neurophysiopathology Techniques and is completing her medical degree. Within her professional competence, she contributed to patient examination assistance, technical acquisition of vestibular and neurophysiological tests, organization of clinical and instrumental data, literature review, conceptual discussion, manuscript preparation, and critical revision.

The clinical interpretation of all vestibular findings and their integration into the diagnostic and rehabilitative framework were performed by Leonardo Manzari, MD.

### 2.4. Case Selection

Four representative cases were selected retrospectively from patients evaluated for persistent dizziness or persistent postural instability after an acute, episodic, or apparently resolved vestibular event. The cases were not selected as a consecutive epidemiological sample and were not intended to support prevalence estimates or statistical generalization.

They were selected because they illustrated distinct and clinically relevant patterns of dissociation between vestibular domains. Specifically, the selected cases represented:

1. low-frequency or integrative vestibular dysfunction without clear peripheral lateralization;
2. incompletely compensated unilateral vestibular asymmetry;
3. selective unilateral otolithic loss with preserved semicircular canal high-frequency responses;
4. bilateral sustained vestibular hypofunction unmasked by an apparently resolved BPPV-like event.

The selection was therefore explanatory rather than epidemiological. The cases were chosen to illustrate how similar symptoms of persistent dizziness may arise from different physiological substrates when interpreted through a multidomain vestibular assessment.

### 2.5. Clinical and Instrumental Vestibular Assessment

All patients underwent detailed clinical history and bedside neuro-otological examination. Bedside assessment included evaluation for spontaneous nystagmus, gaze-evoked nystagmus, positional nystagmus, head-shaking nystagmus, skew deviation when clinically indicated, postural stability, and signs of active benign paroxysmal positional vertigo.

Instrumental assessment was performed according to clinical indication and availability of complete diagnostic data. All instrumental vestibular tests were performed according to standard clinical protocols for each commercially available diagnostic platform and in accordance with the manufacturer’s operating procedures. As this was a retrospective, hypothesis-generating case series derived from routine clinical practice, test paradigms were selected according to clinical indication rather than applied as a fixed experimental protocol. The main devices, testing domains, and clinically relevant protocol information are summarized in Supplementary Table S1, while quantitative parameters relevant to the interpretation of each case are reported in Section 3 and, where applicable, in the figures.

The test battery was designed to explore different vestibular domains rather than to privilege a single test modality. High-frequency/transient vestibular function was assessed mainly using video head impulse testing, including horizontal and vertical canal evaluation and, when available, suppression head impulse paradigm testing. Otolithic function was assessed using cervical and ocular vestibular-evoked myogenic potentials. Sustained or low-frequency vestibular processing was assessed using caloric testing, rotational chair testing, optokinetic stimulation, and other visual–vestibular or velocity-storage-dependent paradigms. Dynamic vestibular asymmetry was assessed using head-shaking testing and skull vibration-induced nystagmus when clinically appropriate. The purpose of this multidomain approach was to compare the relative integrity of transient/high-frequency responses with sustained/low-frequency and integrative vestibular mechanisms. The clinically relevant finding was not considered to be the isolated abnormality of a single test but the pattern of agreement or dissociation across tests [3,4,5,6,7].

### 2.6. Diagnostic Equipment and Testing Domains

Vestibular and audiological assessments were performed using commercially available clinical diagnostic platforms. The equipment used in routine practice included platforms for video-nystagmography, video head impulse testing, caloric stimulation, rotational testing, and vestibular-evoked myogenic potentials.

The main diagnostic platforms and the vestibular domains explored by each test are summarized in Supplementary Table S1. Supplementary Table S1 provides additional methodological detail on the clinical test paradigms used in this case series, including the diagnostic platform, the vestibular domain primarily explored, and the main physiological target of each procedure, thereby supporting reproducibility while preserving the readability of the main manuscript.

The same clinical battery was not applied identically to every patient; tests were selected according to clinical indication and available diagnostic data. The aim was not to compare devices but to interpret established clinical tests according to the dominant vestibular domain interrogated by each paradigm.

This framework was not intended to classify tests as exclusively belonging to one domain. Rather, each test was interpreted according to the dominant physiological dynamics it preferentially interrogates.

### 2.7. Interpretation Framework

Vestibular findings were interpreted within a time-domain framework. High-frequency, high-acceleration, and jerk-dominated stimuli were considered to preferentially probe transient vestibular responses and direct pathway behavior. Low-frequency, sustained, rotational, caloric, and optokinetic paradigms were considered to more strongly engage sustained vestibular processing, visual–vestibular integration, and velocity-storage-dependent mechanisms.

The proposed framework does not claim that caloric or rotational testing are new diagnostic tools. These are established vestibular investigations. The novelty of the present approach lies in the integrated interpretation of established vestibular tests across temporal domains, with particular attention to clinically meaningful dissociations between preserved transient responses and impaired sustained or integrative processing [3,5,6,7,8,9,10].

### 2.8. Statistical Analysis

No inferential statistical analysis was performed because of the small number of illustrative cases and the hypothesis-generating design of the study. The study was not designed to estimate disease prevalence, test diagnostic accuracy, quantify treatment efficacy, or validate a new diagnostic entity. The analysis was descriptive and comparative across cases, focusing on patterns of vestibular-domain dissociation and their possible implications for individualized vestibular rehabilitation.

### 2.9. Ethics and Consent

The study was conducted in accordance with the principles of the Declaration of Helsinki and was approved by the MSA Institutional Review Board, MSA ENT Academy Center, Cassino, Italy (protocol code 003, 8 November 2025).

All clinical data were derived from routine diagnostic assessments performed as part of standard specialist neuro-otological care. Data were retrospectively reviewed and anonymized before inclusion in the manuscript. No experimental intervention was performed for research purposes.

Written informed consent for the anonymized use and publication of clinical information was obtained from all patients included in the case series.

## 3. Results

Four illustrative cases were selected to represent distinct clinical phenotypes of persistent dizziness within the proposed transient–sustained vestibular framework. The cases are presented below to highlight different patterns of agreement or dissociation between transient/high-frequency vestibular responses and sustained/low-frequency, otolithic, visual–vestibular, or velocity-storage-dependent processing.

### 3.1. Case 1: Persistent Dizziness with Preserved High-Frequency Canal Function and Impaired Low-Frequency Integrative Processing

A 71-year-old woman presented with recurrent episodes of positional vertigo associated with persistent imbalance over the preceding month. Her medical history included a previous episode of acute vertigo in 2020, clinically consistent with benign paroxysmal positional vertigo (BPPV). At the time of evaluation, however, she no longer reported true spinning vertigo but rather ongoing instability and motion sensitivity.

Bedside examination was unremarkable. No spontaneous nystagmus was observed in the sitting position, positional testing was negative, and head-shaking did not elicit post-head-shaking nystagmus.

Quantitative vestibular testing showed preserved high-frequency vestibular function. Video head impulse testing demonstrated normal gain values across all semicircular canals, without relevant asymmetry. Horizontal canal gains ranged from 0.95 to 1.09, anterior canal gains from 0.77 to 0.98, and posterior canal gains from 0.85 to 0.98.

In contrast, low-frequency vestibular assessment disclosed abnormalities. Rotational chair testing showed reduced gain, approximately 0.32 at 0.23 Hz, with preserved phase and no relevant directional preponderance. Optokinetic responses were reduced and asymmetric, with mean slow-phase velocity of −8.2°/s for leftward stimulation and +4.6°/s for rightward stimulation at a target velocity of 40°/s.

Skull vibration-induced nystagmus was low in amplitude and not clearly lateralizing. Right-sided stimulation produced a peak slow-phase velocity of approximately 4°/s and mean slow-phase velocity of 1.4°/s, whereas left-sided stimulation produced a peak slow-phase velocity of approximately 2°/s and mean slow-phase velocity of 0.8°/s. No spontaneous or positional nystagmus was instrumentally recorded.

This case illustrates persistent dizziness in the presence of preserved high-frequency semicircular canal function but impaired low-frequency and visual–vestibular integrative responses. In the absence of clear lateralizing peripheral signs, the pattern was interpreted as predominantly sustained-domain or velocity-storage-related dysfunction rather than active BPPV or ongoing unilateral peripheral vestibular deficit.

### 3.2. Case 2: Persistent Postural Instability with Preserved vHIT and Residual Dynamic Vestibular Asymmetry

A 45-year-old man was evaluated for persistent postural instability. His medical history was notable for two remote episodes of probable BPPV during the summer of 2021. No further positional vertigo had occurred thereafter, but the patient continued to complain of chronic disequilibrium and persistent unsteadiness during daily activities.

Bedside examination showed no evidence of active positional vertigo. However, head-shaking elicited right-beating horizontal nystagmus, suggesting residual vestibular asymmetry.

High-frequency vestibular function was largely preserved. On vHIT, horizontal canal gain was 0.93 on the left and 0.99 on the right. Anterior canal gain was also preserved, with values of 0.89 on the left and 0.94 on the right, with only mild reduction in the left posterior canal gain, approximately 0.84. Suppression head impulse paradigm responses were similarly maintained, indicating substantial preservation of high-frequency vestibulo-ocular reflex function.

In contrast, low-frequency and dynamic tests disclosed persistent abnormalities. Rotational chair testing showed reduced sinusoidal gain, approximately 0.51 at 0.23 Hz, with phase of approximately −9°. Post-rotatory time constants were short, approximately 2.6 s and 3.2 s, suggesting reduced low-frequency vestibular responsiveness and limited velocity-storage efficiency rather than a major active canal paresis.

Optokinetic testing was asymmetric, with lower gain during leftward stimulation than during rightward stimulation, approximately 0.50 versus 0.68 at 40°/s. Skull vibration-induced nystagmus was consistently right-beating, more evident during left-sided stimulation, supporting the presence of residual left-sided vestibular asymmetry.

Cervical and ocular vestibular-evoked myogenic potentials were symmetrical, and pure-tone audiometry was not clinically contributory. These findings argued against significant current otolithic or cochlear asymmetry.

This case illustrates persistent postural instability in a patient with preserved or near-preserved high-frequency canal function but residual low-frequency and dynamic vestibular asymmetry. The combination of normal or near-normal vHIT/SHIMP findings with abnormal head-shaking, SVIN, optokinetic asymmetry, and altered rotational chair responses was interpreted as incomplete compensation of a previous left peripheral vestibular hypofunction, mainly expressed in the sustained and integrative domains.

### 3.3. Case 3: Selective Unilateral Otolithic Loss Despite Preserved Semicircular Canal High-Frequency Responses

A 37-year-old man was evaluated 15 days after the abrupt onset of an acute vestibular syndrome characterized by severe vertigo followed by persistent unsteadiness. At bedside examination, he showed a spontaneous left-beating first-degree nystagmus, with a few additional left-beating beats after head-shaking, indicating residual imbalance in vestibular tone.

During diagnostic positioning, a stationary clockwise torsional nystagmus was observed only in the left lateral position, whereas no significant nystagmus was elicited in the right lateral position. Because the positional nystagmus was sustained and non-paroxysmal, it was considered inconsistent with active posterior canal BPPV and more suggestive of positional modulation of residual unilateral vestibular asymmetry.

Video head impulse testing showed largely preserved semicircular canal high-frequency function. Mean lateral canal gains were normal bilaterally, with values of 1.03 on the left and 0.97 on the right. Vertical canal gains were also preserved overall, except for a mild reduction in the right posterior canal gain, approximately 0.87. SHIMP responses were symmetric, with lateral canal values of approximately 0.90 on the left and 0.89 on the right, supporting relative preservation of the canal high-frequency direct pathway.

In contrast, vestibular-evoked myogenic potentials demonstrated marked right-sided otolithic deficit. The oVEMP recorded beneath the left eye was absent, consistent with right utricular dysfunction, and the cVEMP recorded from the right sternocleidomastoid muscle was absent, indicating right saccular dysfunction. In both tests, the asymmetry ratio reached 100%, documenting severe ipsilateral otolithic loss.

Additional findings supported persistence of vestibular asymmetry rather than complete recovery. The combination of spontaneous nystagmus, residual head-shaking nystagmus, and positional modulation of the imbalance suggested that vestibular compensation was still incomplete at the time of evaluation.

This case illustrates a dissociated unilateral vestibular phenotype after acute vestibular syndrome, characterized by preserved semicircular canal high-frequency function, severe right-sided utricular and saccular loss, and incomplete central re-equilibration. It supports the view that normal vHIT does not exclude clinically meaningful unilateral vestibular injury, particularly when the lesion predominantly affects otolithic pathways.

### 3.4. Case 4: Bilateral Sustained Vestibular Hypofunction Unmasked by an Apparently Resolved BPPV-like Event

A 68-year-old woman was evaluated after a recent episode of brief positional vertigo that had spontaneously resolved approximately 8 days before clinical assessment. At the time of evaluation, she no longer reported true spinning vertigo but complained of persistent postural instability and subjective imbalance, particularly during standing and ambulation. No associated auditory symptoms were reported, and neurological examination was unremarkable.

The clinical picture was initially consistent with BPPV-related residual dizziness, namely dizziness following a probable but undocumented episode of BPPV that had resolved spontaneously before specialist evaluation.

Bedside examination did not disclose signs of active positional vertigo. No spontaneous nystagmus was observed, head-shaking was not clearly contributory, and no definite lateralizing signs emerged from bedside assessment.

High-frequency vestibular function was preserved. Video head impulse testing demonstrated normal vestibulo-ocular reflex gain across all semicircular canals, with no relevant corrective saccades, indicating preserved high-frequency canal function. Vestibular-evoked myogenic potentials were also present and symmetrical bilaterally, with an oVEMP asymmetry ratio of approximately 11% and a cVEMP asymmetry ratio of approximately 3%, indicating no significant utricular or saccular asymmetry.

In contrast, low-frequency vestibular testing disclosed a markedly abnormal pattern. Rotational chair testing showed substantially reduced sinusoidal gain, approximately 0.22 at 0.24 Hz, with minimal directional preponderance. Post-rotatory responses were severely reduced, and multi-turn testing showed very short time constants, approximately 2.2 s and 1.0 s, indicating markedly impaired velocity-storage function. This profile was consistent with bilateral low-frequency vestibular hypofunction predominantly affecting the sustained domain.

Additional investigations showed a normal brain MRI, excluding clinically relevant structural central lesions, whereas serum vitamin D level was severely reduced, approximately 4 ng/mL.

This case illustrates a striking dissociation within the vestibular system. High-frequency canal function and transient otolithic responses were preserved, whereas low-frequency rotational responses were profoundly reduced. The clinical picture initially interpreted as residual dizziness after a BPPV-like event was therefore better understood as a condition in which a recent vestibular event unmasked a previously compensated bilateral dysfunction affecting the sustained vestibular domain.

### 3.5. Cross-Case Comparison

The four cases showed different patterns of vestibular-domain dissociation. In all cases, persistent symptoms were not adequately explained by a single test result considered in isolation. Rather, the clinically relevant information emerged from the relationship between transient/high-frequency responses and sustained, otolithic, visual–vestibular, or velocity-storage-dependent findings.

Supplementary Table S2 summarizes the main clinical and physiological features of the four cases.

Overall, the cases support the view that persistent dizziness may arise from different combinations of preserved and impaired vestibular domains. The same clinical complaint may therefore correspond to different physiological substrates, requiring different diagnostic interpretations and different rehabilitation priorities.

**Figure 2:**
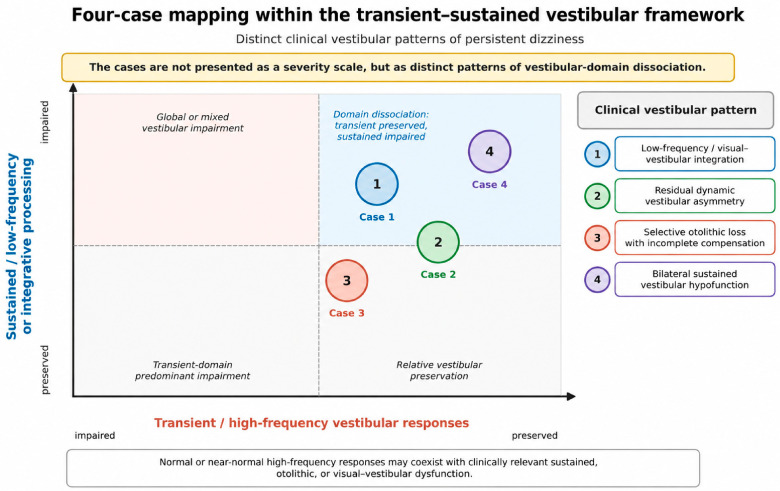
Relative positioning of the four cases within the transient–sustained vestibular framework. The four cases are schematically mapped according to the relative preservation or impairment of transient/high-frequency vestibular responses and sustained/low-frequency or integrative vestibular processing. The figure is not intended to represent a severity scale but rather to illustrate distinct clinical vestibular patterns of persistent dizziness arising from different patterns of vestibular-domain dissociation.

The relative position of the four cases within the transient–sustained vestibular framework is illustrated in Figure 2.

**Figure 3:**
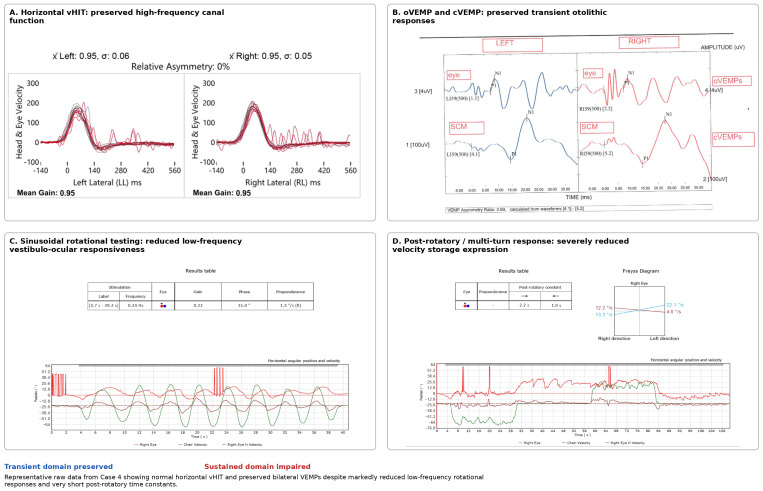
Representative raw data from Case 4 showing dissociation between preserved transient vestibular responses and impaired sustained vestibular processing. (**A**) Horizontal video head impulse test traces showing preserved high-frequency semicircular canal function without relevant corrective saccades. (**B**) Ocular and cervical vestibular-evoked myogenic potentials showing preserved and symmetrical transient otolithic responses. (**C**) Sinusoidal rotational chair testing showing markedly reduced low-frequency vestibulo-ocular reflex gain. (**D**) Post-rotatory/multi-turn rotational responses showing severely reduced time constants, consistent with impaired velocity storage function. Together, these raw data illustrate the core dissociation proposed in this paper: preserved transient vestibular function in the presence of markedly impaired sustained vestibular integration [3,6,8,9,10].

Representative raw data from Case 4 are shown in Figure 3.

## 4. Discussion

The present hypothesis-generating case series illustrates that persistent dizziness may arise from different patterns of dissociation between transient/high-frequency vestibular responses and sustained/low-frequency or integrative vestibular processing. Across the four cases, symptoms were not adequately explained by a single test result considered in isolation. Rather, the clinically relevant information emerged from the relationship between preserved and impaired vestibular domains. This was particularly evident in patients with preserved or near-preserved vHIT responses but abnormal rotational, optokinetic, otolithic, or dynamic asymmetry findings.

The aim of this work was not to introduce new vestibular tests. Caloric testing, rotational chair testing, optokinetic stimulation, VEMPs, vHIT, head-shaking testing, and skull vibration-induced nystagmus are established clinical investigations. The proposed contribution lies instead in the integrated interpretation of these tests according to the temporal domain they preferentially interrogate. In this framework, high-frequency and high-acceleration tests mainly explore rapid vestibular responses, whereas caloric, rotational, visual–vestibular, and post-rotatory paradigms more strongly engage sustained vestibular processing and velocity-storage-dependent mechanisms [3,5,6,7,8,9,10].

### 4.1. Persistent Dizziness as a Multidomain Vestibular Problem

Persistent dizziness is often interpreted as a non-specific residual symptom, particularly after an apparent episode of BPPV or after resolution of an acute vestibular syndrome [1,2]. However, the present cases suggest that persistent symptoms may reflect different physiological substrates. In Case 1, preserved high-frequency semicircular canal function coexisted with reduced rotational gain and abnormal optokinetic responses, suggesting impaired low-frequency or visual–vestibular integration. In Case 2, preserved vHIT/SHIMP responses coexisted with abnormal head-shaking nystagmus, SVIN, optokinetic asymmetry, and altered rotational responses, suggesting incomplete compensation of a previous unilateral vestibular asymmetry. In Case 3, high-frequency canal responses were preserved despite severe unilateral otolithic loss, illustrating that normal vHIT does not exclude clinically meaningful vestibular injury. In Case 4, normal vHIT and symmetrical VEMPs coexisted with markedly reduced rotational gain and very short post-rotatory time constants, suggesting bilateral sustained vestibular hypofunction.

These observations support the view that persistent dizziness should not be approached as a single diagnostic category. Similar symptoms may arise from different combinations of preserved and impaired vestibular domains. A patient with persistent instability after a BPPV-like episode may have residual otolithic dysfunction, incomplete compensation of a unilateral lesion, maladaptive visual dependence, impaired low-frequency vestibular responsiveness, or a previously compensated bilateral vestibular deficit unmasked by a recent event. The clinical challenge is therefore not only to determine whether a test is normal or abnormal but to understand which vestibular domain is failing to reintegrate.

### 4.2. Why Normal vHIT Does Not Exclude Vestibular Dysfunction

The vHIT is a major advance in vestibular diagnostics because it allows for rapid, canal-specific assessment of high-frequency vestibulo-ocular reflex function. Nevertheless, a normal vHIT should not be interpreted as evidence that the entire vestibular system has recovered. The test primarily interrogates high-acceleration, high-frequency canal responses. It does not fully assess low-frequency canal responsiveness, sustained vestibular integration, post-rotatory behavior, visual–vestibular interaction, or the temporal dynamics of velocity storage [3,4,6,8,9,10].

This distinction was central to the present case series. Case 3 showed that a patient may have preserved high-frequency canal responses while presenting severe unilateral utricular and saccular dysfunction. Case 4 showed that preserved high-frequency canal function and symmetrical transient otolithic responses may coexist with markedly reduced low-frequency rotational responses. These dissociations are clinically relevant because the patient’s symptoms may arise from the impaired domain rather than from the domain that is preserved.

The same reasoning applies in the opposite direction. Caloric or rotational abnormalities do not simply replace vHIT findings; rather, they provide complementary information. The clinical value of a multidomain assessment lies in identifying the pattern of convergence or divergence between tests. When the vHIT, VEMPs, caloric testing, rotational testing, OKN, HST, and SVIN are interpreted together, the clinician may better distinguish between active peripheral asymmetry, incomplete compensation, otolithic loss, visual–vestibular maladaptation, and sustained-domain hypofunction.

### 4.3. Velocity Storage as a Clinical Interpretive Construct

Velocity storage is a central mechanism that prolongs and spatially organizes vestibular responses beyond the immediate dynamics of the semicircular canals [6,8,9,10]. It contributes to temporal integration, post-rotatory responses, optokinetic after-responses, gravity-referenced reorientation, and visual–vestibular interaction. From a clinical perspective, velocity storage should not be considered merely as a laboratory concept but as a framework for understanding persistent symptoms that emerge when rapid vestibular reflexes appear preserved but sustained integration remains abnormal.

The present cases do not prove the existence of a new disease entity. Rather, they illustrate how velocity-storage-dependent behavior may help interpret persistent dizziness in selected patients. Reduced rotational gain, shortened time constants, abnormal optokinetic responses, impaired visual–vestibular interaction, and discordance between high-frequency and low-frequency findings may all indicate that sustained vestibular processing remains dysfunctional. In this sense, the term “velocity-storage-related dysfunction” is used here as a hypothesis-generating interpretive construct, not as a validated diagnostic label.

This distinction is important. The proposed framework does not claim that rotational or caloric tests are new, nor does it claim that every patient with persistent dizziness has velocity-storage dysfunction. Instead, it suggests that established vestibular tests may be more clinically informative when interpreted within a time-domain model that distinguishes transient responses from sustained integration [3,5,6].

### 4.4. Relationship with PPPD and the Bárány Society Criteria

The relationship between the present framework and persistent postural–perceptual dizziness (PPPD) deserves specific consideration. According to the Bárány Society consensus criteria, PPPD is a chronic vestibular disorder defined by five required criteria [11]. It is characterized by one or more symptoms of dizziness, unsteadiness, or non-spinning vertigo present on most days for three months or more; these symptoms are exacerbated by upright posture, active or passive motion, and exposure to moving or complex visual stimuli. It is precipitated by conditions that cause vertigo, unsteadiness, dizziness, or balance disturbance; causes significant distress or functional impairment; and is not better accounted for by another disease or disorder.

The present model is not intended to replace PPPD, nor to classify all persistent dizziness as an organic vestibular disorder. Rather, it provides a structured approach to identifying patients in whom persistent symptoms may be better explained, at least in part, by demonstrable vestibular-domain dissociation. This distinction is important. PPPD is not simply a diagnosis of exclusion, and abnormal findings on examination or vestibular testing do not automatically exclude PPPD. However, such findings may indicate an ongoing precipitating condition, a co-existing vestibular disorder, or an incompletely compensated vestibular deficit that should be integrated into the final diagnostic formulation.

Therefore, a patient with persistent dizziness, normal high-frequency vestibular testing, normal caloric and rotational testing, normal VEMPs, normal OKN, no residual vestibular asymmetry, and symptoms fulfilling the Bárány criteria may appropriately be considered as having PPPD. Conversely, when multidomain vestibular assessment reveals persistent abnormalities in otolithic, visual–vestibular, dynamic asymmetry, low-frequency, or velocity-storage-dependent mechanisms, these findings should not be disregarded. In such cases, the clinical picture may represent PPPD coexisting with a residual vestibular disorder, or persistent dizziness primarily driven by a demonstrable vestibular-domain dysfunction.

The proposed time-domain framework should therefore be regarded as complementary to the Bárány PPPD classification. It may help clinicians avoid premature attribution of persistent dizziness to a purely functional disorder when standard high-frequency testing is normal but sustained, otolithic, or visual–vestibular domains remain abnormal. At the same time, it preserves PPPD as an appropriate diagnosis when all Bárány criteria are met and no alternative or coexisting vestibular-domain abnormality better accounts for the patient’s symptoms.

### 4.5. Implications for Targeted Vestibular Rehabilitation

The main clinical implication of this framework concerns rehabilitation. Vestibular rehabilitation should not be prescribed as a generic intervention for all patients with persistent dizziness [12,13,14]. Instead, therapy should be tailored to the physiological domain that appears primarily dysfunctional.

In patients with predominant visual–vestibular or optokinetic abnormalities, rehabilitation may emphasize graded visual-motion exposure, optokinetic stimulation, visual dependence reduction, and visual–vestibular reintegration. In patients with residual dynamic vestibular asymmetry, therapy may focus on gaze stabilization, dynamic balance, habituation to head movements, and progressive recalibration of asymmetrical vestibular tone. In patients with selective otolithic loss, rehabilitation should include gravity-referenced balance training, postural orientation, multisensory reweighting, and exercises emphasizing verticality and body alignment. In patients with bilateral sustained vestibular hypofunction, rehabilitation should prioritize substitution strategies, balance training under reduced sensory conditions, gait stabilization, and carefully graded low-frequency or sustained vestibular stimulation.

This approach does not imply that vestibular rehabilitation must be rigidly determined by one test. Rather, the rehabilitation prescription should emerge from the overall physiological pattern. The goal is to identify which domain is failing: transient canal function, otolithic function, dynamic symmetry, visual–vestibular integration, low-frequency vestibular responsiveness, or velocity-storage-dependent processing. A multidomain test battery can therefore help translate diagnostic physiology into individualized rehabilitation targets [3,12,13,14].

### 4.6. Clinical and Research Implications

The present case series supports a broader clinical message: persistent dizziness should be evaluated with attention to temporal dynamics [5,15]. A patient may recover the high-frequency components of vestibular reflex function while remaining impaired in sustained or integrative domains. Conversely, a patient may have no active positional nystagmus but still show residual vestibular asymmetry or low-frequency hypofunction. These patterns may explain why some patients continue to experience instability despite apparently reassuring bedside or high-frequency test findings.

From a research perspective, this framework should be tested prospectively. Future studies should include larger cohorts, predefined inclusion criteria, standardized vestibular test batteries, quantitative comparison between transient and sustained vestibular measures, and longitudinal rehabilitation outcomes. Multicenter validation will be essential to determine whether the proposed patterns are reproducible across different clinical settings and whether domain-specific rehabilitation improves outcomes compared with standard vestibular rehabilitation [12].

### 4.7. Limitations

This study has several limitations. First, it is based on four illustrative cases and was not designed to estimate prevalence, diagnostic accuracy, or treatment efficacy. The cases were selected for their explanatory value and do not represent a consecutive epidemiological sample. Second, no inferential statistical analysis was performed because the study was hypothesis-generating and case-based. Third, although the cases were assessed using a multidomain vestibular approach, the same test battery was not applied identically to every patient; tests were selected according to clinical indication and available diagnostic data. Fourth, the proposed framework has not yet been externally validated in other centers. Fifth, rehabilitation implications were derived from physiological interpretation rather than from a controlled treatment-outcome protocol.

These limitations are inherent to the conceptual and case-based nature of the study. The present manuscript should therefore be interpreted as a clinical hypothesis-generating framework rather than as a validated diagnostic or therapeutic model. Its purpose is to provide a structured way of interpreting persistent dizziness through time-domain dissociation and to encourage prospective studies capable of testing this approach systematically.

## 5. Conclusions

Persistent dizziness after an apparently resolved vestibular event should not be interpreted automatically as a non-specific residual symptom or as a purely functional disorder. The present hypothesis-generating case series illustrates that similar clinical complaints may arise from different patterns of dissociation between preserved transient/high-frequency vestibular responses and impaired sustained/low-frequency, otolithic, visual–vestibular, or velocity-storage-dependent processing.

The proposed framework does not introduce new vestibular tests and does not claim to validate a new diagnostic entity. Rather, it provides a structured way to integrate established vestibular investigations within a time-domain model. In this approach, normal or near-normal vHIT findings should not be considered sufficient to exclude clinically meaningful vestibular dysfunction, particularly when symptoms persist and other vestibular domains remain unexplored.

The clinical relevance of this framework lies in its potential to guide individualized vestibular rehabilitation. By identifying the dominant dysfunctional domain, clinicians may better tailor therapy toward visual–vestibular integration, residual dynamic asymmetry, otolithic dysfunction, bilateral sustained hypofunction, or velocity-storage-related impairment.

Given the small number of illustrative cases and the absence of external validation, these observations should be regarded as preliminary and hypothesis-generating. Prospective studies with larger cohorts, standardized multidomain vestibular protocols, longitudinal rehabilitation outcomes, and multicenter validation are required to determine the diagnostic and therapeutic value of the proposed time-domain framework.

## Data Availability

The data presented in this study are not publicly available due to privacy and ethical restrictions related to clinical patient information. Anonymized data may be made available from the corresponding author upon reasonable request and in accordance with applicable ethical and privacy regulations.

## Supplementary Materials

The following supporting information can be downloaded at: https://www.mdpi.com/article/10.3390/healthcare14111560/s1, Supplementary Table S1: Diagnostic equipment and vestibular domains assessed in the present case series; Supplementary Table S2: Cross-case comparison of clinical phenotype, vestibular-domain dissociation, and rehabilitative implications.

## Abbreviations

The following abbreviations are used in this manuscript:

BPPV	benign paroxysmal positional vertigo
cVEMP	cervical vestibular-evoked myogenic potential
HIMP	head impulse paradigm
HST	head-shaking test
OKN	optokinetic nystagmus
oVEMP	ocular vestibular-evoked myogenic potential
PPPD	persistent postural–perceptual dizziness
SHIMP	suppression head impulse paradigm
SVIN	skull vibration-induced nystagmus
VEMP	vestibular-evoked myogenic potential
vHIT	video head impulse test
VNG	videonystagmography
VOR	vestibulo-ocular reflex
VORS	vestibulo-ocular reflex suppression
VVOR	visually enhanced vestibulo-ocular reflex
VSI	velocity storage integrator
VSM	velocity storage mechanism
