# Supplementary material for: Persistent Dizziness and Time-Domain Dissociation in Vestibular Function: A Hypothesis-Generating Case Series and Spatiotemporal Framework for Targeted Vestibular Rehabilitation
Source: Healthcare (Basel). 2026 Jun 3;14(11):1560. doi: 10.3390/healthcare14111560 (PMC13256592; doi:10.3390/healthcare14111560)
Supplement: Supplementary file 1 [file healthcare-14-01560-s001.zip › healthcare-4350260-supplementary.pdf]

Table S1. Diagnostic equipment and vestibular domains assessed in the present case series.

Domain	Test/paradigm	Equipment/platform used	Main physiological target
**Transient/high-frequency canal domain**	vHIT; HIMP; SHIMP; VVOR; VORS	ICS Impulse vHIT, Natus / Otosuite Vestibular	High-acceleration angular VOR; direct pathway; overt/covert saccades; visual–vestibular interaction during high-frequency head stimulation
**VNG / oculomotor / visual–vestibular domain**	Spontaneous nystagmus; positional testing; OKN; gaze; smooth pursuit; skew deviation	ICS Dizcovery VNG, Natus / Otosuite Vestibular	Oculomotor control; visual–vestibular integration; fixation-related responses; central ocular motor signs
**Caloric domain**	Air caloric testing	ICS Aircal, Natus / Otosuite Vestibular	Low-frequency horizontal canal responsiveness; unilateral weakness; sustained vestibular asymmetry
**Rotational / sustained vestibular domain**	Pendular rotational testing; sinusoidal rotational testing; post-rotatory responses; velocity-storage-related paradigms	Nystalyze VNG module Inventis/Synapsys rotational chair	Low-frequency VOR gain; phase; post-rotatory responses; time constants; velocity-storage efficiency
**Otolithic domain**	cVEMP; oVEMP	ICS Chartr EP 200, Natus	Saccular and utricular pathways; transient otolithic responses; asymmetry ratio
**Dynamic vestibular asymmetry**	HST; SVIN	VNG/video-Frenzel recording and clinical skull vibrator Inventis	Residual vestibular asymmetry; dynamic imbalance; vibration-induced responses

Abbreviations: cVEMP, cervical vestibular-evoked myogenic potential; HIMP, head impulse paradigm; HST, head-shaking test; OKN, optokinetic nystagmus; oVEMP, ocular vestibular-evoked myogenic potential; SHIMP, suppression head impulse paradigm; SVIN, skull vibration-induced nystagmus; vHIT, video head impulse test; VNG, video-nystagmography; VOR, vestibulo-ocular reflex; VORS, vestibulo-ocular reflex suppression; VVOR, visually enhanced vestibulo-ocular reflex.

Table S2. Cross-case comparison of clinical phenotype, vestibular-domain dissociation, and rehabilitative implications.

Case	Clinical phenotype	Preserved domain	Impaired domain	Dominant interpretation	Rehabilitation focus
Case 1	Persistent dizziness after previous BPPV-like episode	Transient/high-frequency canal function preserved on vHIT	Sustained low-frequency and visual–vestibular integration impaired; reduced rotational gain and abnormal OKN	Predominant low-frequency integrative or velocity-storage-related dysfunction	Visual–vestibular integration, graded optokinetic exposure, and sustained vestibular recalibration
Case 2	Chronic postural instability after remote probable BPPV episodes	High-frequency canal function preserved or near-preserved on vHIT/SHIMP	Dynamic and sustained-domain asymmetry; abnormal HST, SVIN, OKN asymmetry, and reduced rotational responses	Incomplete compensation of previous left vestibular asymmetry	Rehabilitation targeting residual asymmetry, dynamic balance, gaze stability, and reintegration of sustained responses
Case 3	Persistent unsteadiness after acute vestibular syndrome	Semicircular canal high-frequency responses preserved on vHIT/SHIMP	Severe right otolithic deficit on cVEMP/oVEMP; residual spontaneous and head-shaking nystagmus	Selective right otolithic deficit with incomplete compensation	Otolithic-oriented rehabilitation, gravity-referenced balance training, postural control, and multisensory reweighting
Case 4	Persistent imbalance after apparently resolved BPPV-like event	High-frequency canal function and transient otolithic responses preserved	Markedly reduced rotational gain and very short post-rotatory time constants	Bilateral sustained vestibular hypofunction unmasked by recent event	Bilateral vestibular loss strategies, substitution, balance training, and low-frequency/sustained stimulation

Abbreviations: BPPV, benign paroxysmal positional vertigo; vHIT, video head impulse test; SHIMP, suppression head impulse paradigm; HST, head-shaking test; SVIN, skull vibration-induced nystagmus; OKN, optokinetic nystagmus; cVEMP/oVEMP, cervical/ocular vestibular-evoked myogenic potential.
